# DNA Damage Focus Analysis in Blood Samples of Minipigs Reveals Acute Partial Body Irradiation

**DOI:** 10.1371/journal.pone.0087458

**Published:** 2014-02-03

**Authors:** Andreas Lamkowski, Fabien Forcheron, Diane Agay, Emad A. Ahmed, Michel Drouet, Viktor Meineke, Harry Scherthan

**Affiliations:** 1 Institut für Radiobiologie der Bundeswehr in Verb. mit der Universität Ulm, München, Germany; 2 Institut de Recherche Biomédicale des Armées (IRBA), Bretigny sur Orge, France; University of Science and Technology of China, China

## Abstract

Radiation accidents frequently involve acute high dose partial body irradiation leading to victims with radiation sickness and cutaneous radiation syndrome that implements radiation-induced cell death. Cells that are not lethally hit seek to repair ionizing radiation (IR) induced damage, albeit at the expense of an increased risk of mutation and tumor formation due to misrepair of IR-induced DNA double strand breaks (DSBs). The response to DNA damage includes phosphorylation of histone H2AX in the vicinity of DSBs, creating foci in the nucleus whose enumeration can serve as a radiation biodosimeter. Here, we investigated γH2AX and DNA repair foci in peripheral blood lymphocytes of Göttingen minipigs that experienced acute partial body irradiation (PBI) with 49 Gy (±6%) Co-60 γ-rays of the upper lumbar region. Blood samples taken 4, 24 and 168 hours post PBI were subjected to γ-H2AX, 53BP1 and MRE11 focus enumeration. Peripheral blood lymphocytes (PBL) of 49 Gy partial body irradiated minipigs were found to display 1–8 DNA damage foci/cell. These PBL values significantly deceed the high foci numbers observed in keratinocyte nuclei of the directly γ-irradiated minipig skin regions, indicating a limited resident time of PBL in the exposed tissue volume. Nonetheless, PBL samples obtained 4 h post IR in average contained 2.2% of cells displaying a pan-γH2AX signal, suggesting that these received a higher IR dose. Moreover, dispersion analysis indicated partial body irradiation for all 13 minipigs at 4 h post IR. While dose reconstruction using γH2AX DNA repair foci in lymphocytes after *in vivo* PBI represents a challenge, the DNA damage focus assay may serve as a rapid, first line indicator of radiation exposure. The occurrence of PBLs with pan-γH2AX staining and of cells with relatively high foci numbers that skew a Poisson distribution may be taken as indicator of acute high dose partial body irradiation, particularly when samples are available early after IR exposure.

## Introduction

The growing application of ionizing radiation (IR) in clinical therapeutic procedures and radiation accidents are major sources of human IR exposure. Irradiation accident patterns fall largely into the category of partial body irradiation (PBI) with an inhomogeneous field distribution, while homogeneous total body irradiation occurred only rarely in reported radiation accidents [2], [3]. In terms of clinical treatment decisions, it is crucial to rapidly obtain an estimate of the doses and exposure scenario to predict the expected severity of radiation-induced damages. Biodosimetry tools can provide dose estimations that are needed to predict the clinical courses and to prepare for medical treatments and resources. However, these assays often require days until results are available [4], [5]. Comparisons of the biological consequences of radiation exposure in different tissues on the same individual are scarce, even in animal models. Here we made use of the minipig large animal model to investigate the suitability of the γH2AX DNA damage focus assay to detect PBI. In the last decade, the Göttingen minipig model was established as a clinical model for radiation injuries of the cutaneous [1], [6], [7], the hematopoietic radiation syndrome [8] and for biodosimetry [9].

One prominent feature of the exposure to IR is the formation of DNA double strand breaks (DSBs) that threaten a cell's survival. DSB-elicited phosphorylation of histone H2AX occurs at serine 139 in H2AX molecules (γ-H2AX) surrounding the DSBs, primarily by the ATM kinase [10]. γH2AX serves as a platform for the DNA damage response that signals DSB formation and directs DNA repair (e.g., [11], [12]). Immunostaining of γH2AX [13] and other factors of the DNA damage response at focal sites surrounding DSB sites has led to the establishment of the γH2AX focus assay in peripheral blood cells [14], [15]. The γH2AX focus assay has been shown to disclose IR exposures and provide information on DSB formation in various exposure scenarios (e.g., [16]–[19]).

Numerous *in vitro* studies highlighted the validity of the γH2AX assay for rapid detection of ionizing radiation exposure and absorbed doses in biological dosimetry [20]–[22]. *In vivo* animal studies with murine [23] and non-human primates [5], [24] have investigated radiation-induced DSB and focus formation after whole and partial body irradiation. Such studies exploited lymphocytes by virtue of minimal-invasive sample recovery from exposed individuals. However, little is known about the suitability of the γH2AX assay to reveal the absorbed doses after an acute high dose PBI event. Recent multi lab comparisons have revealed that the γH2AX assay faces limitations when protracted exposures are addressed [25].

Here, we used the Göttingen minipig model established for cutaneous radiation syndrome (CRS) treatment options [1], [6] to study the DNA damage response of peripheral lymphocytes recovered after high dose PBI of an approx. 125 cm^2^ lumbar region of the minipig back and investigated the DNA damage and its repair in lymphocytes of thirteen ^60^Co γ-irradiated minipigs applying γH2AX, 53BP1 and MRE11 immunostaining of blood samples obtained 4, 24 and 168 hours after gamma irradiation with an average dose of 49 Gy (±6%). The time points studied were defined by the treatment plan of the CRS wound healing study in the minipig model [6], and compared to the previously characterized the DNA damage response in keratinocytes of acutely 50 Gy-exposed minipig skins [18]. In all, our results corroborate those of Moroni et al. suggesting that the minipig is a potent model for γH2AX biodosimetry [9].

## Results

### DSB-associated foci in partial-body γ-irradiated minipigs

**Figure 1:**
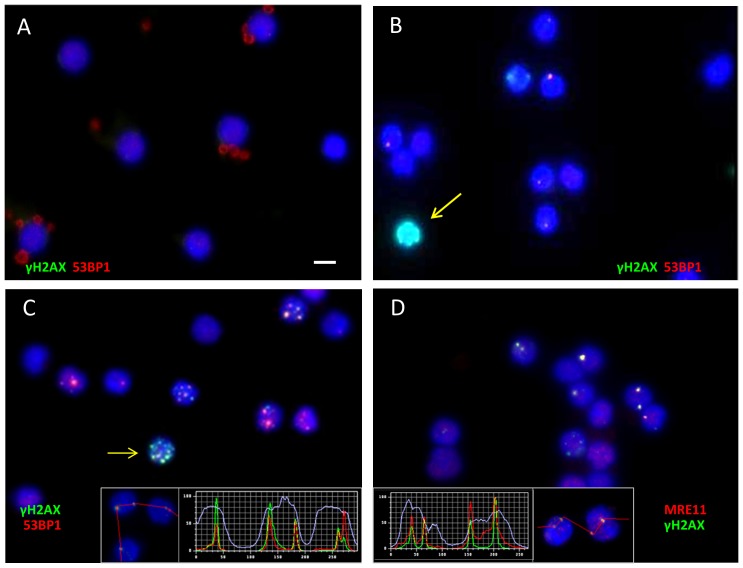
Images showing foci of γH2AX, 53BP1 and MRE11 DSB-marking proteins in nuclei of peripheral blood lymphocytes of minipigs. (**A–C**) Images of minipig PBL immunostained for γH2AX (green), 53BP1 (red) and nuclei (DNA, blue; DAPI). (**A**) Control cells without IR exposure and DNA damage foci. (**B**) Minipig PBL 4 h post 49 Gy irradiation revealing fluorescent foci of γH2AX and 53BP1 marking DSBs. Foci usually contain variable amounts of both DSB markers leading to reddish – greenish colored foci due to color overlap (see insets in C,D). A cell with pan-γH2AX staining shows heavy overall green labeling (arrow). (**C**) PBL nuclei obtained 4 h post IR. A cell with 8 foci is seen in the image center (arrow). Inset: fluorescence intensity profiles across 5 foci in 3 PBL nuclei revealing colocalization of green γH2AX and red 53BP1 fluorescence intensity peaks. (**D**) PBLs of a 4 h sample showing colocalization of the DNA repair protein MRE11 (red) and γH2AX (green) at DNA damage foci, those appear whitish due to color overlap. The inset shows fluorescence intensity profiles across 4 foci in 2 PBL nuclei revealing colocalization of green γH2AX and red MRE11 fluorescence intensity peaks. Magnification 630× in the original micrographs.

**Figure 2:**
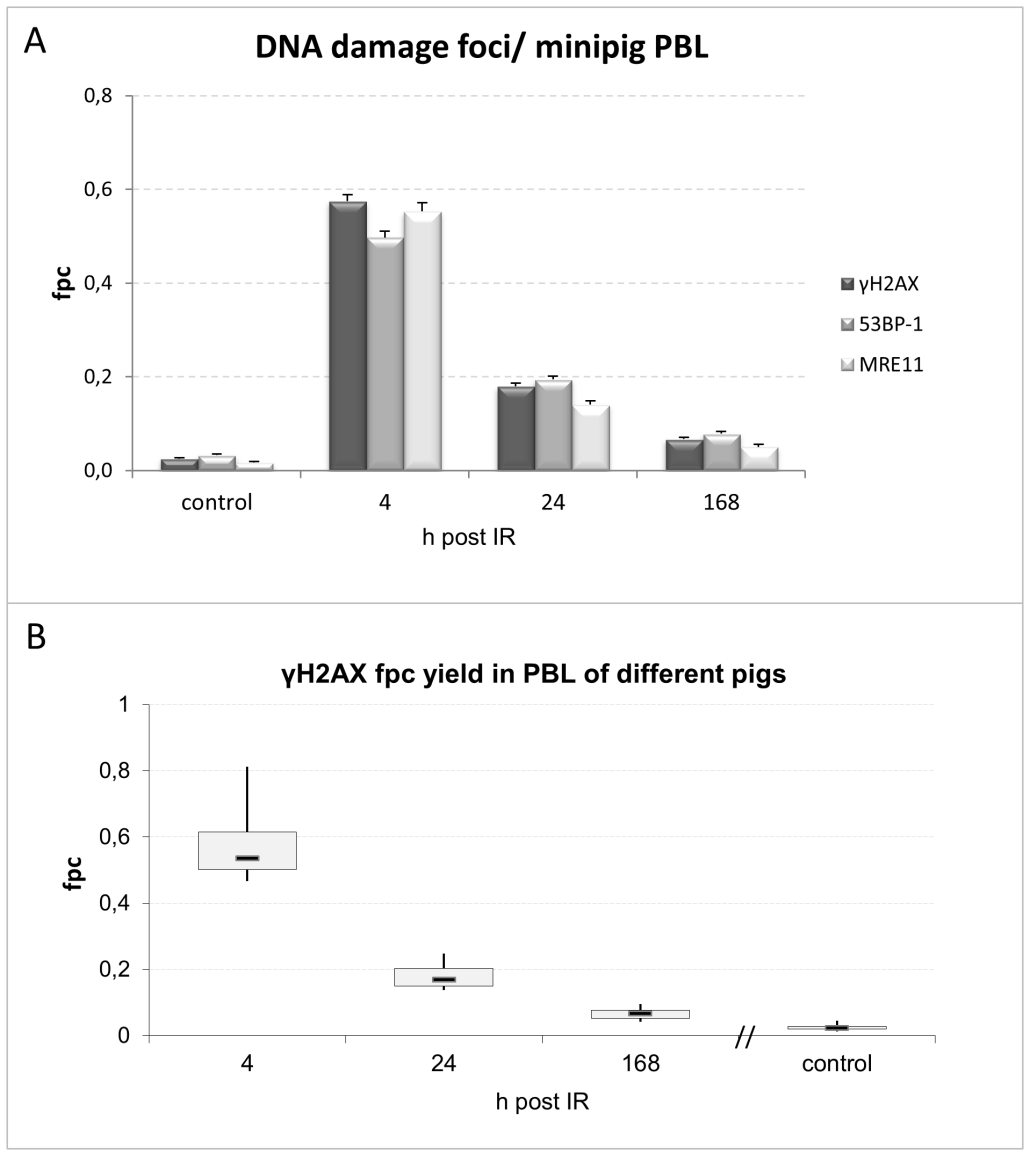
Mean γH2AX, 53BP1 and MRE11 foci numbers in peripheral blood lymphocytes from 13 minipigs detected in control and blood samples obtained 4, 24 and 168 h post IR. (A) Foci per cell values are greatly increased 4 h after 49 Gy partial body exposure and decline with passing time. Error bars represent SE. (B) Box plot showing variation of the different γH2AX fpc values for the 13 minipigs. Whiskers represent minimum and maximum values; a box comprises 50% of the data points.

Acute partial body exposure at the minipig lumbar region with in average 49 Gy of Co-60 γ rays induced DSBs in nuclei of peripheral blood lymphocytes (PBL). DSBs were visualized by staining for γH2AX foci that, upon microscopic analysis, correlate with DSB numbers in the low dose range [17], [26], [27]. Furthermore, we investigated radiation-induced foci of the DNA damage sensor protein 53BP1 [28], [29] and the DNA repair protein MRE11 that is a component of the DNA damage response involved in DSB recognition and repair [11], [30], [31]. The average frequency of DNA damage (γ-H2AX, 53BP1) and repair protein (MRE11) foci in minipig peripheral blood lymphocytes was determined by manually investigating immunostained PBL of 13 partial body irradiated minipigs before (control) and 4 h, 24 h and 168 h after partial body irradiation (Figs. 1, 2) of a ∼125 cm^2^ region of the minipig back. This γ-irradiation scheme [1] resulted in a delivered mean dose of 49 Gy (±6%; Table 1) at the skin surface facing the radiation source.

**Table 1:** Mean γH2AX fpc values in control and post IR minipig PBL.

Minipig	control	4 h	24 h	168 h	dose [Gy] at entry area
1	0.03	0.60	0.15	0.07	50.5
2	0.03	0.61	0.14	0.10	48.8
3	0.03	0.49	0.19	0.05	49.6
4	0.02	0.49	0.20	0.09	49.6
5	0.05	n.s.	0.21	0.04	47.1
6	0.04	0.51	0.25	0.05	44.5
7	0.03	0.51	0.23	0.08	47.4
8	0.02	0.51	0.17	0.08	46.3
9	0.02	0.47	0.20	0.08	47.2
10	0.02	0.56	0.15	0.08	48.1
11	0.02	0.64	0.15	0.06	49.2
12	0.02	0.72	0.14	0.06	50.1
13	0.01	0.81	0.16	0.05	50.6

n.s., sample not available.

Immunofluorescent staining of mononuclear peripheral blood cells revealed in control samples an average of 0.03 γH2AX foci per cell (fpc) (±0.0023 SE). Gamma irradiation induced significantly increased average γH2AX foci values 4 hours post irradiation (0.57 fpc±0.0134; Fig. 2). Both, 53BP1 and MRE11 foci showed colocalization with γH2AX (Fig. 1B–D), as confirmed by overlapping fluorescence intensity profiles at foci (Fig. 1C,D). In average there were 0.5 fpc for 53BP1 (±0.0133 SE) and 0.55 MRE11 fpc (±0.0186) 4 hours post irradiation (Fig. 2; Tables 1, 2, 3). At more advanced time points post IR there was a drop of the mean fpc number in all pigs, indicating progression of DNA repair (Fig. 2; Tables 1, 2, 3). Twenty-four hours post IR the average foci numbers in lymphocytes of thirteen pigs were 0.18 fpc (±0.0064) for γH2AX, 0.19 fpc for 53BP1 (±0.0075) and 0.14 MRE11 fpc (±0.0082). 168 hours post IR there were 0.07 γH2AX fpc (±0.0041), 0.08 53BP1 fpc (±0.0049) and 0.05 MRE11 fpc (±0.005) (Fig. 2A), with the fpc values still being highly significantly elevated above control (p<0.0001).

**Table 2:** Mean 53BP1 fpc values in control and post IR minipig PBL.

Minipig	control	4 h	24 h	168 h
1	0.04	0.51	0.15	0.05
2	0.04	0.44	0.16	0.08
3	0.03	0.46	0.26	0.08
4	0.02	0.48	0.21	0.10
5	0.07	n.s.	0.28	0.07
6	0.04	0.48	0.21	0.06
7	0.04	0.46	0.25	0.09
8	0.03	0.42	0.16	0.07
9	0.04	0.42	0.23	0.10
10	0.02	0.49	0.15	0.09
11	0.03	0.53	0.16	0.08
12	0.02	0.54	0.13	0.07
13	0.01	0.72	0.17	0.07

n.s, sample not available.

**Table 3:** Mean MRE11 fpc values in control and post IR minipig PBL.

Minipig	control	4 h	24 h	168 h
1	0.02	0.57	0.11	0.12
2	0.03	0.75	0.11	0.10
3	0.03	0.50	0.18	0.05
4	0.02	0.44	0.16	0.05
5	0.02	n.s.	0.12	0.04
6	0.02	0.54	0.21	0.03
7	0.02	0.53	0.21	0.02
8	0.01	0.49	0.19	0.06
9	0.01	0.51	0.12	0.02
10	0.02	0.49	0.09	0.06
11	0.01	0.65	0.13	0.04
12	0.02	0.57	0.11	0.05
13	0.01	0.63	0.12	0.04

n.s, sample not available.

### High dose *in vivo* partial body irradiation induces fpc variation

**Figure 3:**
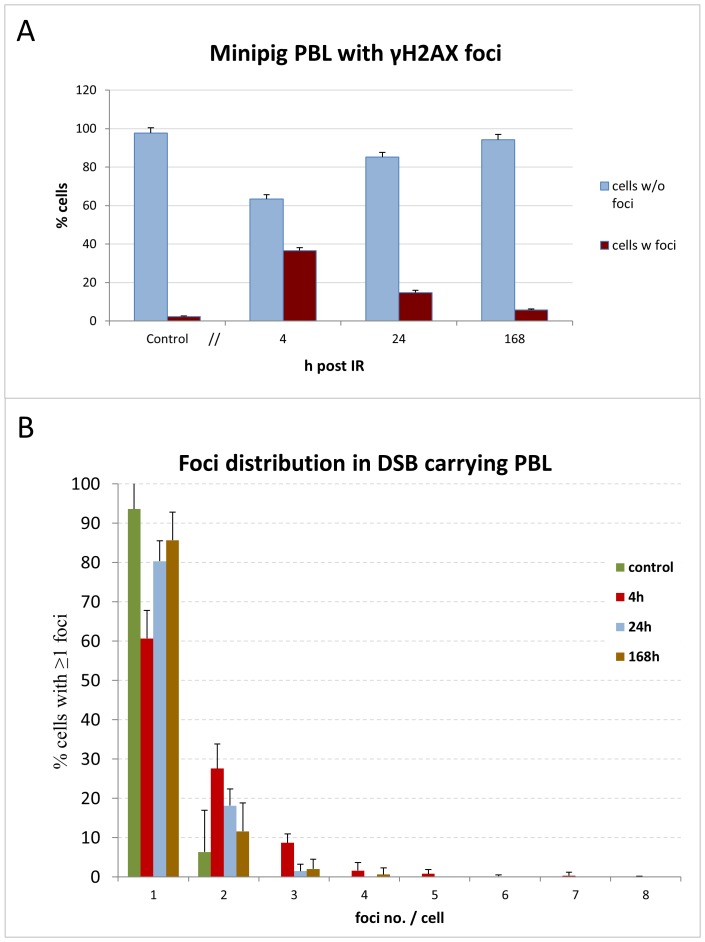
Distribution of γH2AX foci in minipig PBL samples. (A) Average frequency (%) of PBL cells without (w/o) and with (w) γH2AX foci. While in controls nearly all cells were without foci, cells displaying foci were significantly increased 4, 24 and 168 h post IR; differences amongst all time points were highly significant (p<0.0001). (B) Average foci numbers in DSB-positive PBL nuclei. Average frequency (%) of foci-expressing PBL with ≥1 γH2AX focus in 13 investigated minipigs before (control) and 4, 24, and 168 h after PBI. In controls most γH2AX-positive nuclei showed 1 focus, while at 4 h post IR numerous cells contained 2 or 3 foci, with a few cells showing up to 8 foci. Cells with higher foci numbers are absent at later time points. Error bars represent SD.

The acute ∼49 Gy γ exposure of the minipigs was achieved after about 82 minutes of irradiation. In control samples nearly all cells were without DNA damage foci (Fig. 3A), while 4 hours post irradiation only 64% of PBL cells were without foci. Foci-negative cells had increased again to 85% and 94% in samples obtained 24 h and 168 h post IR, respectively (Fig. 3A). Investigating the foci-carrying cells more closely revealed that the exposure scheme led to the formation of 1–8 foci in DSB-carrying cells (Fig. 1C; 3B) in 37% of PBL 4 h post IR (Fig. 3A). Nuclei exhibiting 1 fpc were the major fraction in all pig samples studied, and cells endowed with 5–8 foci per nucleus were only observed in samples obtained 4 h post irradiation (Fig. 1C, 3). Samples collected 24 and 168 h post IR displayed cells with a maximum at 3 or 4 foci per nucleus, respectively (Fig. 2, 3B). Neglecting focus loss thru DNA repair in the first 4 h, these values suggest that at least 37% of PBL transited through the exposed lumbar skin area during the 82 minute γ radiation exposure.

### DNA Repair progression in minipig lymphocytes

**Figure 4:**
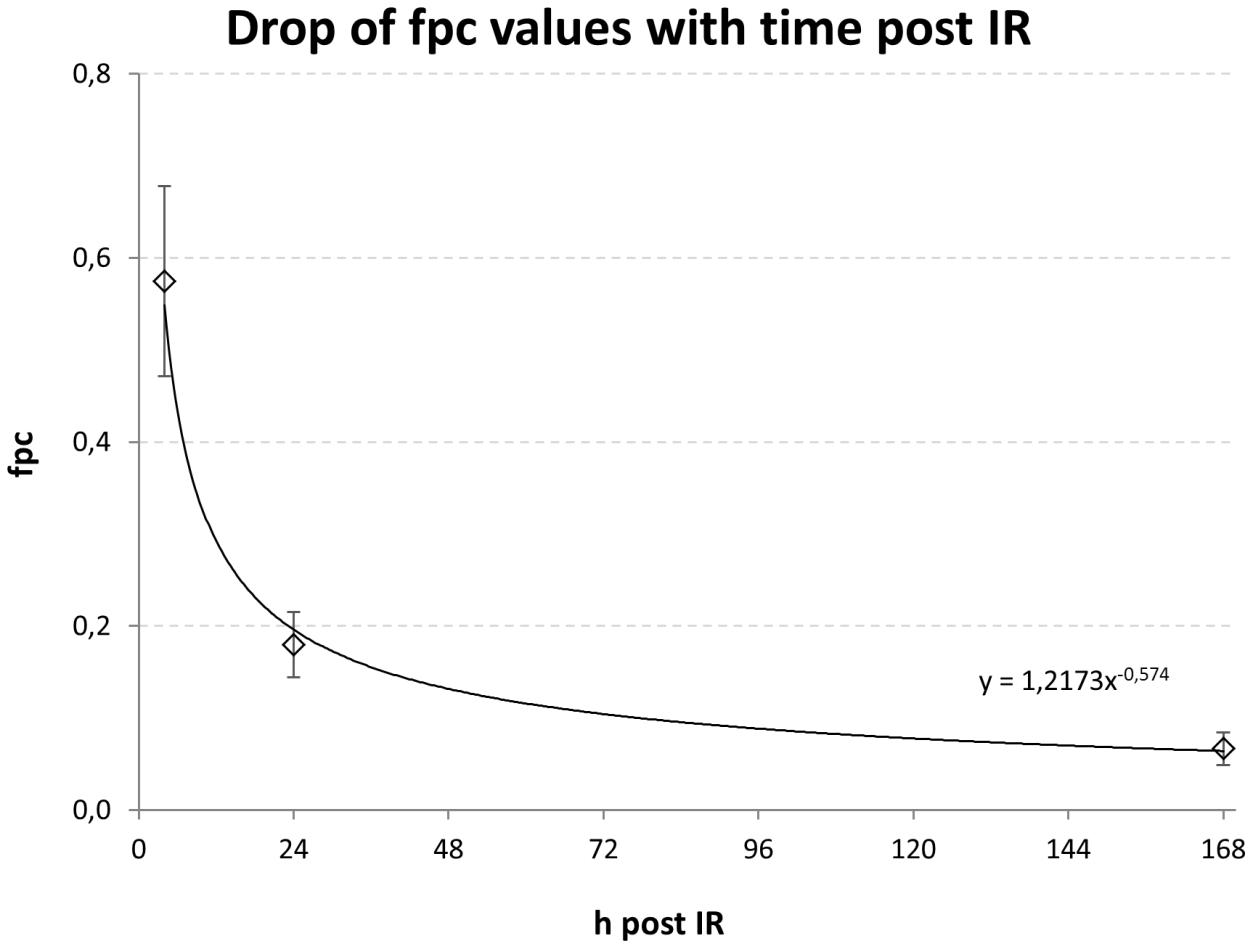
Decline of mean fpc values in PBL of ∼49 Gy PBI γ-exposed minipigs. Mean fpc values ± SD are shown. While the study comprises only 3 time points the shape of the obtained function is consistent with that known for DNA repair after *in vitro* irradiation of minipig cells [9].

Next we estimated the repair capacity of the different animals to obtain an overview of individual variation. Four hours post IR 37% of all cells carried DSBs, while at 168 h post IR 5.7% of cells exhibited foci. Plotting the fpc values over time resulted in an exponential function (Fig. 4) that suggests that 68% of DSBs (foci) present at 4 h were removed by DNA repair in the following 20 h while 12% of the DSBs present at 4 h remained unrepaired even after 168 h, possibly due to the presence of complex DNA lesions at these sites. While our data comprise only three time points post IR, they still reveal a typical biphasic course of DNA repair with slower repair kinetics after 24 hours, with the standard deviations of the mean fpc values of the time course (Fig. 4) suggesting individual dose responses and DNA repair capacities among the thirteen minipigs.

### Dispersion analysis reveals partial body irradiation

**Figure 5:**
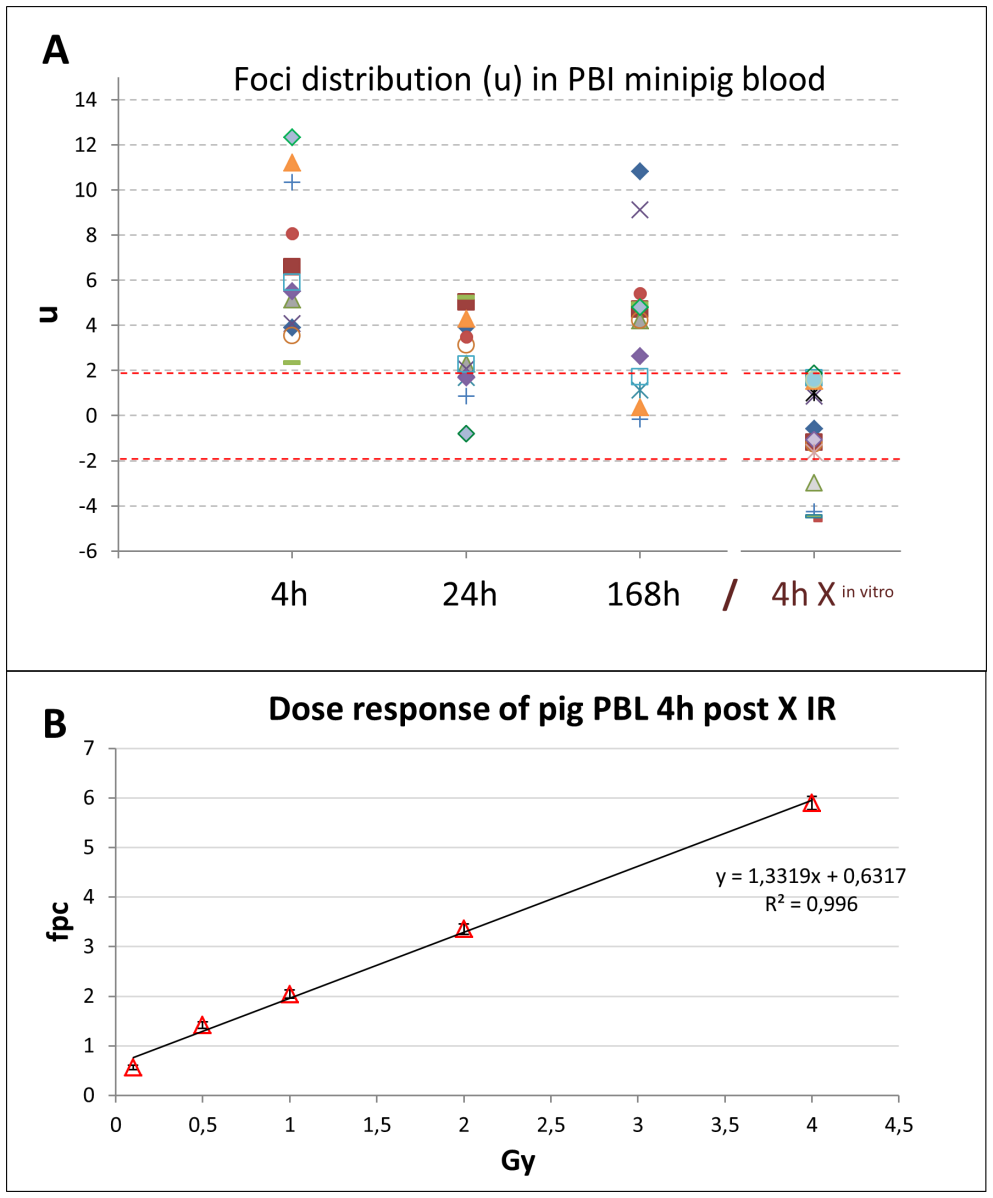
Dispersion analysis (u) of foci distribution in 13 minipig samples at the time post IR indicated. (A) U values outside the red dotted lines indicate over- and underdispersion, which is seen for all values 4 h post IR. *In vitro* 240 kV X ray irradiation of peripheral pig blood (4 h X in vitro) with 0.1, 0.5, 1, 2 and 4 Gy revealed a Poisson distribution for foci in 11 out of 15 samples of three pigs. (B) A linear dose response was obtained for X-irradiated PBL from three pigs obtained 4 h post IR. Mean fpc values (± SE) from three pigs are sown (900 cells per time point).

Since PBI is expected to lead to overdispersion (u) of scored foci/aberration values [21], [32], we checked whether the obtained foci distributions deviate from a Poisson distribution following the contaminated Poisson method of estimation [33]. Assuming that u values <−1.96 and >1.96 reflect under- or overdispersion, a deviation of the γH2AX foci yield from Poisson distribution was detected in all of the irradiated samples (13 pigs) at 4 hours post IR (Fig. 5A), indicating partial body irradiation. At 24 h and 168 h post IR overdispersion was still detected in 69% of samples (Fig. 5A).

To compare with a more homogeneous irradiation scenario we also investigated *in vitro* 240 kV X-irradiated PBL of the domestic pig for deviations from a Poisson distribution. Six different blood samples of three pigs each were irradiated *in vitro* with 0.1–4 Gy 240 kV X rays, fixed and immunostained for γH2AX foci 4 hours post IR. While there was a linear dose response (Fig. 5B), 11 out of 15 samples followed a Poisson distribution. In contrast, all *in vivo* samples 4 h post PBI showed overdispersion (Fig. 5A). The variations observed in the u values of the *ex vivo* irradiated samples may be attributable to inhomogeneity of the X irradiation field (i.e., 2–4%) and individual radiosensitivity, since 3 out of the 4 underdispersed *in vitro* samples stemmed from pig #2.

In the *in vivo* group u variations could relate to deviations in the absorbed doses (49 Gy±6%; Table 1). Furthermore, immunostaining variables like antibody fidelity, cell fixation, etc., may also induce variations in focus yield (for a review see, [34]). Altogether, such variations may lead to insufficient statistical power to reveal deviations from a Poisson distribution, as has been noted for irradiated human lymphocytes [35]. Furthermore, minipig peripheral blood samples of later time points only contained a few PBLs with more than 2 fpc. Thus, the u values were sensitive to small fluctuations in >2 foci carrying cells, which may explain why some pigs failed to show an overdispersion (e.g., pigs no. 3,6,9) at 24 h and 168 hours post IR. Still, dispersion analysis can disclose PBI, even of a limited body surface area, provided blood samples are obtained early after the radiation incident.

### Heavily damaged PBL occur shortly after high dose partial body irradiation

**Figure 6:**
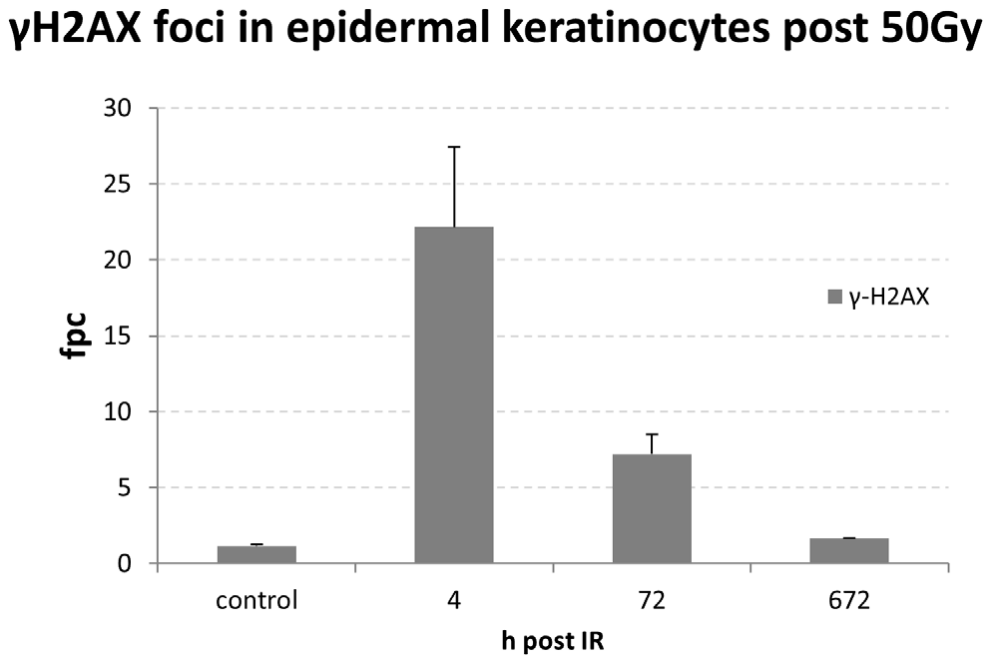
Mean γH2AX fpc values in keratinocytes of the minipig skin facing the beam 4 h after 50 Gy Co-60 γ irradiation. Average of three minipigs (± SD) is shown. Keratinocytes 4 h post IR display >20 γH2AX foci/cell (data taken from [18]).

**Figure 7:**
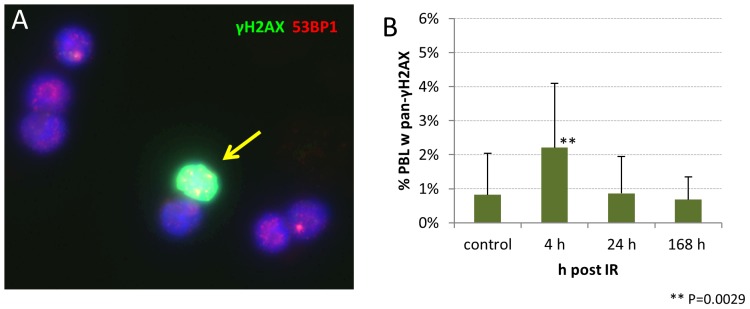
Frequency of pan-γH2AX labeled PBL nuclei in minipig blood. (A) Image of γH2AX (green) and 53BP1 (red) stained PBL showing a pan-γH2AX-positive nucleus (arrow) with numerous 53BP1 (red) foci. DNA is stained in blue (DAPI). (B) Percentage of minipig PBL carrying a pan-γH2AX signal (compare also Fig. 1B). Mean and SD of samples from 13 minipigs is shown.

Comparing the fpc values of minipig lymphocytes with those from skin keratinocytes of the directly irradiated regions in three pigs 4 h post IR (Fig. 6), revealed a distinct difference among IR-induced focus numbers in peripheral blood lymphocytes and the epidermal keratinocytes facing the entry area of the beam, with the latter exhibiting up to 22 fpc (Fig. 6) and most keratinocytes showing pan-γH2AX labeling of the nucleus [18]. Investigation of our non-exposed PBL samples also revealed lymphocytes that exhibited a global pan-nuclear γ-H2AX signal (Fig. 7) at a low percentage (average 0.82%±1.21, SD). However, 4 h post IR there was a highly significantly elevated frequency (P<0.0029) of nuclei with a pan-γH2AX signal (2.21%±1.88%; Fig. 7). The more advanced time points post ∼49 Gy gamma IR showed pan-γH2AX positive nuclei in the range of the control values, i.e. 0.87% (±1.08%) at 24 hours and 0.68% (±0.67% SD) at 168 hours post IR (Fig. 7). These data reveal the presence of severely irradiated pan-γH2AX positive PBL early after *in vivo* acute high dose PBI, suggesting that these cells may have spent a longer time in the irradiated region or may have experienced repeated exposure to absorb a saturating dose. Severely damaged pan-γH2AX–expressing cells may be subject to cell death [18], [36], suggesting that their systemic clearance leads to control values at the later time points.

## Discussion

Partial body exposure of thirteen Göttingen minipigs with acute doses of γ rays at the lumbar body surface led, in peripheral blood lymphocytes, to significant increase of DSB-related nuclear foci numbers 4 h post exposure. Average fpc values decreased with advancing time, but were still significantly elevated above control values seven days post IR. A similar recession of the fpc values over time suggests a comparable repair capacity in the pigs analyzed. The presence of significantly elevated foci numbers of dsDNA damage (γH2AX), sensor (53BP1) and repair (MRE11) proteins in PBL days after the acute IR incident suggests an ongoing DNA damage response at chromatin regions that likely contain complex DNA lesions that are difficult to repair (e.g., [18], [37]). Additionally, persistence of γH2AX after DSB rejoining at such nuclear regions may also be considered [38], [39].

Our foci data in minipig PBL are in agreement with a previous minipig study that applied the same PBI conditions and showed significantly elevated levels of γH2AX foci in PBL of three pigs 25 days post irradiation [1], and the data of Moroni et al. who noted the persistence of elevated fpc values in 3.8 and 5 Gy whole body irradiated minipigs 10 days post IR [9]. On the other hand, keratinocyte nuclei of directly 50 Gy-exposed epidermal regions of three minipigs in average displayed in average 22 γH2AX fpc 4 h post IR [18], which contrasts with the significantly lower average fpc numbers (∼0.5 fpc) in lymphocytes of the same pigs. The differences in average fpc numbers between PBL and keratinocytes in the same animals may be attributed to different exposure conditions - circulating blood cells will absorb reduced doses likely due to a shorter traversal time of the irradiation field, while the resident skin cells will absorb the entire regional dose applied. In agreement, keratinocytes at the entrance area of the γ beam in average accumulated 49 Gy over 82 minutes of irradiation and showed a saturated pan-γH2AX signal in most nuclei 4 h post IR. Most lymphocytes, on the other hand, will have passed the irradiated tissue volume in a shorter time, as is indicated by 1–8 foci in γH2AX-positive PBL in similarly irradiated minipigs 4 h post IR. The observed range of DSB foci could also have been influenced by variations in the staining protocol [25], [34] and the systemic elimination of lethally irradiated blood cells. The latter is supported by the occurrence of a significantly elevated frequency of pan-γH2AX-positive nuclei 4 h post IR. PBL with pan-H2AX phosphorylation may represent cells in the early stages of cell death [18], [36] that are subject to systemic clearance. The latter may have led to the absence of such cells from blood samples taken 24 h or 168 h post IR. Alternatively, a possible recovery through DNA repair may also be considered. However, in light that pan-γH2AX keratinocyte nuclei at 4 h post IR still displayed >22 fpc at 20 h post IR [18] and the fact that such PBL were not detected, renders recovery of pan-γH2AX PBL by DNA repair unlikely.

It has been noted that the majority of accidental radiation exposures involved inhomogeneous or partial body irradiation [2], [21]. Currently, it is still challenging to derive an accurate dose assessment after PBI and to eliminate the influence of varying energy depositions in irradiated body volumes while performing biodosimetry [40], [41]. Previous cytogenetic studies have revealed that the induction of dicentric chromosomes by partial body irradiation failed to meet a Poisson distribution and tended to be overdispersed [21], [24], [32], [42], [43] because recirculation of lymphocytes from non-affected organs will produce a mixed PBL population after PBI. Based on the consideration that PBI induces such a mixed population *in vivo*, the contaminated Poisson method [33] represents an approach to assess foci distribution patterns. Rothkamm et al. (2007) applied dispersion analysis to γH2AX foci in PBL of patients who underwent CT scans and obtained u values suggesting overdispersed γH2AX fpc distributions that met with the partial body exposures studied [16]. Evaluating the obtained γH2AX foci distributions in our acutely *in vivo* minipig samples using the contaminated Poisson method revealed an overdispersion of foci scores 4 h after IR in all investigated PBL samples. This agrees with overdispersion of γH2AX foci distribution observed after a single 2 Gy PBI exposure in head and neck cancer radiotherapy patients [19]. At the more advanced time points post IR ∼30% of minipig samples again showed a Poisson distribution. DNA repair, inhomogeneities in the irradiation field and immunostaining variables may also lead to changes of focus yield and to insufficient statistical power [34], [35]. While storage and transport conditions may also induce variation, these are controlled for in most experimental settings. Furthermore, our minipig PBL samples of the advanced time points contained only a few PBLs showing more than 2 fpc, which made the u values sensitive to small fluctuations in cells carrying more foci, potentially explaining the overdispersion in some pigs at 24 h and 168 hours post IR. It appears that dispersion analysis can indicate PBI of a limited body surface area, provided sampling is done early after the radiation incident.

The irradiated minipig lumbar region comprised approx. ∼125 cm^2^ and >600 cm^3^. At a dose rate of 0.6 Gy/min γ-irradiation lasted for about 82 min to achieve the average dose of 49 Gy. A dose response curve obtained with *in vitro* X irradiated lymphocytes of three domestic pigs revealed in average 2 γ-H2AX foci per Gy at 4 hours post 240 kV X irradiation. Using this yield as a surrogate for minipig PBL, one may deduce that 8 foci-displaying *in vivo*-irradiated PBL will have absorbed approximately 4 Gy. At the applied gamma dose rate of 0.6 Gy/min this would translate to an approx. 7 minute transit time of the irradiated lumbar region for these cells. The absorbed dose after 82 min of irradiation was in average 15 Gy at the exit area, which translates to a dose rate of 0.19 Gy/min with an expected ∼2.5 fpc in cells that traversed this region. Shorter transit time and tissue variables such as capillary density, DSB repair progression during the first 4 h post IR (or during 6 h20 when a PBL made only one early transit through the irradiated volume), may have created the frequently encountered 1 focus-containing PBLs. Although bystander effects can also induce DSBs in non-irradiated cells [44]–[46], they seem unlikely in this case, since this requires transcriptional activation of quiescent PBLs [47]. Finally, geometric attributes, as well as the location and size of irradiation fields [42] have to be taken into account when evaluating PBI effects using γH2AX biodosimetry.

In all, our data are in line with a recent report that validates the minipig as a potent model for γH2AX biodosimetry [9]. Further analyses are required to determine the effects of exposure time and dose rate on focus yield in various radiation situations, especially in high dose scenarios. It appears that in PBI scenarios dose reconstruction represents a challenge when it shall be derived from γH2AX foci values of peripheral blood lymphocytes. Notwithstanding, the γH2AX DNA damage focus assay is a potent and rapid bioindicator of irradiation exposure. The occurrence of PBLs with pan-γH2AX staining and of cells with relatively high foci numbers that skew a Poisson distribution may be taken as indicator of acute high dose partial body irradiation, especially when samples are available early after IR exposure. Individuals with such foci patterns shall be recommended for further clinical monitoring.

## Materials and Methods

### Animal model

The current study includes 13 female Göttingen minipigs weighting around 20 kg. The experimental animals were accommodated in separate cages (21±1°C, 55% relative humidity, 12 hour/12-hour light-dark schedule). The minipigs were fed twice a day with solid food and drank water ad libitum. All animal trials were approved by the Animal Ethics Committee of the French Armed Forces Biomedical Research Institute (N°2008/24.0). All pigs were treated in compliance with the French legislation related to animal care and protection.

### Experimental *in vivo* irradiation

The experimental radiation Göttingen minipig model of this study was used as described by Agay et al. [1]. Animal anesthesia was achieved with Tiletamine and Zolazepam (6 mg/kg intramuscularly, Zoletil 100; Virbac, Carros, France) and 1.5% isoflurane (Isoflurane Belamont; Mundipharma, Issy-les-Moulineaux, France). The narcotized minipigs were positioned in a homogeneous horizontal gamma radiation field generated by a ^60^Co source (IRDI 4000; Alstrom, Levallois-Perret, France) and focused to a limited body area in order to ensure a standardized partial body exposure. A lead shield delimitated the radiation exposure to a 125 cm^2^ lumbar skin area and avoided direct irradiation to other organ systems. The lumbar region facing the source was irradiated with an average local surface dose of 49 Gy γ-rays at a dose rate of 0.6 Gy/min, while the opposing region received a local dose of ∼15 Gy [1]. The dose delivered was controlled by an ionization chamber and several 1.5 cm×5 mm Al powder dosimeters (Desmarquest alumina [Al_2_O_3_], Desmarquest Fine Ceramics, France) fixed to the regions traversed by the beam. The doses delivered to the region facing the source (region “A” in Fig. 2 of [1]) are listed in table 1. Peripheral blood samples from 13 pigs were drawn before IR and 4 hours, 24 h and 168 h post irradiation, fixed in Ethanol and stored or transported at −20°C until analysis [48]. Blood samples were obtained from 13 minipigs during a period spanning nearly two years, with minipigs being irradiated in groups of 2 to 4 animals. Isolated white blood cells were stored at −20°C until staining [48].

### *In vitro* X irradiation

Blood samples of 3 domestic pigs were collected in Heparin-containing collection tubes (Saarstedt) at a local slaughterhouse, transferred to the institute (30 min) and irradiated at room temperature with 240 kV X rays (YXLON Maxishot; Hamburg, Germany) filtered with 3 mm beryllium. Absorbed dose was measured with a PTW Unidos dosimeter (PTW Freiburg GmbH, Germany). The dose rate was 1 Gy/min at 13 mA. Control cells were sham-irradiated. Cells were incubated for 4 h in a tissue culture incubator. Lymphocytes were isolated by Ficoll-paque centrifugation (GE healthcare), followed by fixation in ice-cold 70% ethanol and storage at −20°C for at least two days before immunostaining [48].

### Lymphocyte isolation and staining of DNA double-strand break associated foci

Blood samples were taken from anesthetized pigs before (control) and 4 h, 24 h and 7 days post irradiation. Lymphocytes were isolated by Ficoll-paque (GE healthcare) density centrifugation followed by ethanol fixation and subjected to immunofluorescent staining to detect DNA damage-associated protein accumulation as microscopic foci at DNA double-strand break sites as described [48]. We applied primary antibodies against γ-H2AX, 53BP1 and MRE11 and detected them with secondary goat anti-mouse Alexa-488 (Table 4). 53BP1 and MRE11 primary antibodies were detected using donkey anti-rabbit Cy3-labeled antibodies (Table 4). The number of irradiation-induced DNA damage and repair protein foci was analyzed by an experienced investigator in lymphocyte nuclei (n>300 PBL/sample) by manual focus counting directly in a Zeiss Axioimager 2i fluorescence microscope. Cells which showed deformed nuclei or were overlapping were excluded from analysis. Minute foci scattered over the nucleus and the cytoplasm were considered as background and excluded from enumeration. Images were recorded using the ISIS fluorescence imaging system (MetaSystems). Fluorescence intensity profiles were obtained using the profile option of the ISIS imaging software. For each pig and time point 300 (in some cases 400) cells were analyzed by fluorescence microscopy from at least three independent staining experiments/sample. The average values of the different time points were derived from 5200 and 4800 (4 h, were one pig sample was lacking) evaluated cells per pig group.

**Table 4:** Antibodies, dilutions and sources.

Antibodies	Supplier	Order no.	Dilution
*Primary antibodies*			
Mouse anti-γ-H2AX	Millipore	JBW301	1/250
Rabbit anti-53BP1	Acris	NB 100-304	1/500
Rabbit anti- hMRE11	Acris	NB 100-142	1/100
*Secondary antibodies*			
Goat anti-mouse Alexa-488	Mobitec	A11017	1/500
Donkey anti-rabbit-Cy3 Fab	Dianova	711-167-003	1/1000

### Statistics

Data are presented as mean with corresponding standard error SE = SD/√(n-1), unless otherwise stated. The Student's t-test was used to compare the obtained focus values.
